# Comparing the Documentation of Phosphatidylethanol and Alcohol History in Hypertension Management in Primary Care: A Cross-Sectional Study

**DOI:** 10.1177/21501319261465137

**Published:** 2026-07-02

**Authors:** Anders Norrman, Åsa Thurfjell, Maria Hagströmer, Johanna Adami, Lena Lundh, Jan Hasselström

**Affiliations:** 1 Division of Family Medicine and Primary Care, Department of Neurobiology, Care Sciences and Society, 27106 Karolinska Institutet, Huddinge, Sweden; 2 Academic Primary Health Care Centre, Region Stockholm, Stockholm, Sweden; 3 Division of Physiotherapy, Department of Neurobiology, Care Sciences and Society, 27106 Karolinska Institutet, Huddinge, Sweden; 4 25548 Sophiahemmet University, Stockholm, Sweden

**Keywords:** alcohol drinking, blood pressure, glycerophospholipids, hypertension, prevention, primary care, screening

## Abstract

**Introduction/Objectives:**

Hazardous alcohol use is a modifiable risk factor for hypertension, yet it is often underrecognized in primary care, and the role of Phosphatidylethanol (PEth) testing in documenting alcohol use remains unclear. This study assessed alcohol use documentation using PEth and alcohol history in relation to blood pressure (BP) control and patient characteristics, and examined hazardous alcohol use by both methods in relation to BP control.

**Methods:**

All patients with hypertension from a Swedish primary care centre, routinely using PEth, were included in a cross-sectional study based on electronic medical records. Patients were categorized as having controlled (&lt;140/90 mmHg), uncontrolled (≥140/90 mmHg), or apparent treatment-resistant hypertension (aTRH) (≥140/90 mmHg despite ≥3 antihypertensive drugs). Alcohol documentation and hazardous use were analyzed using PEth 16:0/18:1 values and alcohol history (free-text entries or AUDIT). Group differences were examined using chi-square and one-way ANOVA.

**Results:**

A total of 1,826 patients were included (mean age 71 years; 51.5% women); 35% had controlled hypertension, 50% uncontrolled hypertension, and 16% aTRH. Documentation patterns differed by BP control, sex, age, and comorbidities. PEth was documented more often than alcohol history (56.0% vs. 39.9%, respectively). Women, older individuals, and patients with diabetes more frequently lacked documentation. Hazardous alcohol use was identified in 18.4% using PEth and 7.1% using alcohol history, with no differences across BP control groups. Hazardous use was higher in men by PEth, not alcohol history.

**Conclusion:**

PEth testing can complement alcohol history in hypertension management, providing clinically relevant information, although its impact on detecting hazardous drinking or improving BP control remains uncertain.

## What Is Known About the Topic

• Hypertension affects over one billion people globally, yet many individuals fail to achieve target blood pressure levels despite effective pharmacological treatment.
• High alcohol consumption is a modifiable risk factor for hypertension, yet it is often underrecognized in primary care.
• PEth is a sensitive and specific biomarker for recent alcohol intake, increasingly used in healthcare, including hypertension management.

## What This Study Adds

• A description of the documentation of alcohol, with PEth and/or alcohol history, after implementing PEth testing in routine hypertension management in a primary care setting.
• Elevated PEth levels can be found in patients with controlled hypertension, uncontrolled hypertension, and apparent treatment resistant hypertension.
• The findings reveal demographic disparities in alcohol documentation, with older patients and women being less likely to be assessed with PEth and/or alcohol history.

## Introduction

Hypertension affects over one billion people globally and is a significant risk factor for cardiovascular diseases.^1^ Many individuals with hypertension do not achieve target blood pressure (BP).^2,3^ Although effective pharmacological treatment, only one-fifth of individuals with hypertension achieve BP levels below 140/90 mm Hg.^3^ High alcohol consumption elevates BP and increases the risk of developing hypertension.^4^ Treatment guidelines for elevated BP and hypertension suggest a maximum of 100 grams of pure alcohol per week, although the optimal approach is to avoid alcohol entirely.^2^ However, high alcohol consumption often goes unidentified in primary care, making it challenging to manage and treat effectively.^5^ Hazardous alcohol use can be identified through unstructured questioning, screening tools such as the Alcohol Use Disorders Identification Test (AUDIT)^6^ and biomarkers such as Phosphatidylethanol (PEth).^7^ PEth is a direct alcohol biomarker with high sensitivity and specificity as its formation requires alcohol.^7^ PEth concentration in blood rises with repeated exposure and decreases slowly with abstinence, reflecting alcohol intake over the past 2–4 weeks.^8^

The use of PEth is increasing in health care.^9^ In a recent study conducted in Swedish primary care,^10^ PEth levels were analyzed in relation to BP control among patients with hypertension. A higher prevalence of elevated PEth levels was observed among patients with poor BP control despite treatment with three or more antihypertensive agents.^10^ These patients were also assessed using the AUDIT questionnaire; however, this method identified only a subset of patients with elevated PEth levels.^10^ In addition, qualitative studies indicate that general practitioners (GPs) perceive PEth as a valuable tool for alcohol assessment in different clinical situations^11^ including hypertension management.^12^ However, there is a lack of studies examining how PEth is utilised in primary care settings, and whether factors such as age, sex, and comorbidities influence the decision to employ PEth testing.

This cross-sectional, real-world study sought to assess the documentation of alcohol consumption based on PEth results and alcohol history, defined as either free-text entries or AUDIT scores, among patients with hypertension at a primary care centre where PEth testing is routinely implemented in hypertension management. The study aimed to describe alcohol consumption documented through PEth and alcohol history in relation to BP control, sex, age, and comorbidities. Furthermore, the proportion of patients with hazardous alcohol use based on PEth and alcohol history was analyzed in relation to BP control. In addition, gender-specific proportions of hazardous alcohol use, derived from both PEth and alcohol history, were examined.

## Methods

### Design and Setting

This cross-sectional study included 1,826 patients with hypertension from one primary care centre in a Swedish city with approximately 53,000 inhabitants. As of the data collection date, 12,637 patients were registered with the centre. Data from the period December 1, 2021, to November 30, 2023, were collected from electronic medical records (EMR) on January 24, 2024. The average Care Need Index (CNI)^13^ for the centre was 1.18 in 2023.

At the centre, routine PEth testing was introduced in 2016 to enhance lifestyle interventions in hypertension care. Due to low response rates with AUDIT, PEth was adopted without a formal protocol and ordered routinely with standard hypertension laboratory tests, except in patients over 80 years of age. PEth was also used in mental health assessments, sick-leave evaluations, 50-year health checks, and when clinically indicated. Routine testing in diabetes care was discontinued due to low yield. Overall, the exact indications and contexts for PEth testing cannot be fully delineated, as its use was not protocol-driven but rather reflects clinical decisions made by physicians in a real-world primary care setting. No standardized procedure governed patient information. GPs made individual decisions, and PEth was omitted if patients declined. Elevated PEth values (>0.2 μmol/L) were followed up with individualized interventions.

### Sample

**Figure 1.**
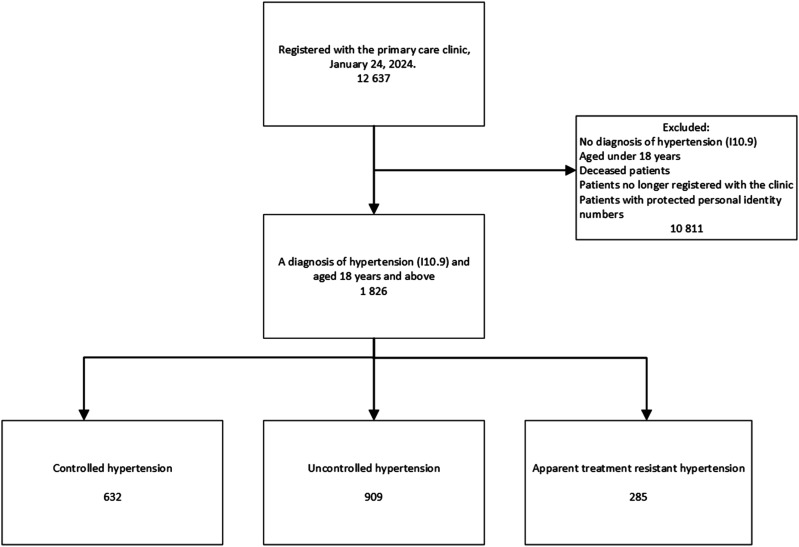
Flowchart of inclusion process and study population divided into three strata: Controlled hypertension < 140/90 mmHg, Uncontrolled hypertension ≥ 140/90 mmHg, and Apparent treatment-resistant hypertension ≥ 140/90 mmHg with at least three classes of antihypertensive medications regardless of class

Patients registered at the centre aged 18 years and older with a documented diagnosis of hypertension (ICD-10 code I10.9) in the EMR were eligible for inclusion. Exclusion criteria included deceased patients, patients no longer registered with the centre, and those with protected personal identity numbers. Study participants were stratified into three categories of hypertension: controlled (<140/90 mm Hg), uncontrolled (≥140/90 mm Hg), and apparent treatment-resistant hypertension (aTRH) (≥140/90 mm Hg with at least three classes of antihypertensive medications regardless of class).^14^ Stratification was based on the most recent BP reading and antihypertensive prescriptions recorded in the EMR at their last visit. It was not specified whether the most recent BP was from an office BP measurement, home BP monitoring, or ambulatory BP monitoring.^2^ Data on the exact prescribed dose, dispensation, and adherence to medication were not available. A flow chart of the selection process is provided in Figure 1.

### Procedure

Data were extracted from the EMRs using the Medrave M4 tool.^15^ The dataset included systolic and diastolic BP, free-text alcohol entries, AUDIT scores, PEth 16:0/18:1 ^9^ values, age, sex, prescribed antihypertensive medications, Body Mass Index (BMI), estimated Glomerular Filtration Rate (eGFR), low density lipoprotein (LDL), and smoking status. Comorbidities (ICD-10 code); diabetes (E10-E14), coronary heart disease (I20-I25), atrial fibrillation (I48), congestive heart failure (I50), and cerebrovascular injury (I61-I64), were also extracted.

Analysis of PEth 16:0/18:1 was performed in EDTA whole blood using liquid chromatography–tandem mass spectrometry (LC–MS/MS)^16^ at an external laboratory utilized by the centre.

In the dataset, PEth values, free-text entries, AUDIT scores, and BP measurements may have been documented at different times during the study period. All data from the EMR, may have been recorded multiple times during the study period. When variables were recorded more than once, the most recent value was used, as only the latest measurement was available in the dataset extracted for analysis using Medrave4.^15^ All data were anonymized before being shared with the research team.

### Definitions of Variables and Hazardous Alcohol Use

Documentation of alcohol consumption in the EMR was defined as the presence of a PEth value, alcohol history (free-text entries or AUDIT scores), both PEth and alcohol history, or no documentation. All individuals’ EMR data were reviewed to determine whether and how alcohol use had been documented. Patients were classified based on the presence or absence of a PEth value and alcohol history (free-text entries or AUDIT scores) in the EMR. They were categorized into four groups: “PEth”, “Alcohol history”, “PEth and Alcohol history”, and “No documentation”.

Hazardous alcohol use based on PEth was defined as PEth ≥0.12 μmol/L (86 ng/mL), as this threshold has been shown, in a population cohort examining alcohol consumption, to identify approximately 90% of individuals who consume more than three alcohol units (12 grams of pure alcohol) per day.^17^

Alcohol history was derived from either AUDIT scores or free-text entries describing alcohol consumption. A total of 829 free-text entries were independently reviewed by two authors (AN and ÅT). Discrepancies in classification were identified in 20 entries and were resolved through discussion. Inter-rater agreement was assessed using Cohen’s kappa coefficient (κ = 0.97). The categorization was based on Swedish national guidelines^18^ for hazardous alcohol use: 10 or more standard units (12 grams of pure alcohol per unit) per week or 4 or more units on the same occasion once a month or more often, aligning with international definitions.^19^ Examples of alcohol history interpreted as hazardous use included free-text entries like “drinking to relieve anxiety”, “clear overconsumption”, or “medication for controlled consumption”. AUDIT scores were originally planned for separate analysis, but only 79 complete scores were available. They were therefore incorporated into the alcohol history categorization (0–7 = no hazardous use; 8–40 = hazardous use).^6^ Thus, the categorisation of alcohol history was based on either free-text entries or AUDIT scores. If both free-text entries and AUDIT scores were available, the categorisation was made based on the free-text.

### Statistical Analysis

Continuous variables were calculated as means with 95% confidence intervals. Categorical variables were calculated as proportions. Comparisons between groups were analysed using one-way ANOVA for means and Chi-square test for categorical data. The proportion of individuals with documented alcohol habits were analyzed in relation to three BP control groups, sex, age, and comorbidities, using contingency tables and Chi-square tests. The proportions of patients with hazardous use, based on PEth and alcohol history respectively, were analysed in relation to BP control groups by contingency tables and chi-square tests. Sex-specific proportions of hazardous alcohol use, derived from both PEth and alcohol history, were analyzed by contingency tables and chi-square tests. A p-value of less than 0.05 was considered significant. Data were analysed with IBM SPSS Statistics Version 26.

### Ethics

The study was approved by the Swedish Ethical Review Authority in Gothenburg (registration numbers 2020–04725 and 2023-02430-02). All data were extracted from EMRs and fully anonymized prior to analysis. The study was conducted in accordance with the principles of the Declaration of Helsinki.

## Results

The characteristics of the study population are described in Table 1. A total of 1,826 patients with a diagnosis of hypertension were included. The mean age was 71 years (SD ± 13), with 51.5% women. Among the study population, 35% had controlled hypertension, 50% had uncontrolled hypertension, and 16% aTRH. Significant differences were observed across the BP control groups in terms of age, BMI, BP, prescribed antihypertensive drugs, comorbidities, LDL, and eGFR.

**Table 1.** Characteristics of Study Population With Controlled Hypertension, Uncontrolled Hypertension, and Apparent Treatment Resistant Hypertension (2021-2023)

​	Controlled hypertension^1^	Uncontrolled hypertension^2^	Apparent treatment resistant hypertension^3^	Total	P-value
N (%)	626 (34.6)	909 (49.7)	285 (15.6)	1,826	​
Age, years (mean ± SD)	72 ± 13	70 ± 14	72 ± 12	71 ± 13	0.00^a^
Women, %, (n)	52.4 (331)	51.6 (469)	49.5 (141)	51.5 (941)	0.72^b^
BMI, kg/m^2^ (mean ± SD)	27.0 ±3.8	28.9 ±4.9	30.3 ± 5.3	28.4 ±4.7	0.01^a^
Systolic blood pressure, mm Hg (mean ± SD) (n)	125 ± 10 (632)	155 ± 16 (793)	156 ± 17 (262)	144 ± 20 (1,689)	0.00^a^
Diastolic blood pressure, mm Hg (mean ± SD)	76 ± 9 (632)	91 ± 11 (793)	90 ± 12 (262)	85 ± 13 (1,689)	0.00^a^
Antihypertensive drugs (mean ± SD)	1.73 ± 0.93	1.38 ± 0.62	3.17 ± 0.39	1.78 ± 0.95	0.00^a^
Diabetes mellitus,^4^ % (n)	27.8 (176)	19.3 (175)	32.6 (93)	24.3 (444)	0.00^b^
Coronary artery disease,^5^ % (n)	21.8 (138)	12.5 (114)	18.2 (52)	16.6 (304)	0.00^b^
Atrial fibrillation,^6^ % (n)	19.0 (120)	11.2 (102)	18.9 (54)	15.1 (276)	0.00^b^
Congestive heart failure,^7^ % (n)	14.6 (92)	7.5 (68)	10.2 (29)	10.4 (189)	0.00^b^
Cerebrovascular injury,^8^ % (n)	10.9 (69)	5.6 (51)	9.8 (28)	8.1 (148)	0.00^b^
Current smoker, % (n)	9.8 (39)	10.6 (53)	8.6 (16)	10.0 (108)	0.31^b^
LDL, mmol/L (mean ± SD)	2.5 (550)	2.9 (703)	2.6 (237)	2.7 (1.490)	0.00^a^
eGFR < 60 mL/min/1,73 m^2^, % (n)	66.1 (621)	68.8 (856)	65.0 (272)	67.3 (1.749)	0.00^a^

BMI; Body Mass Index, LDL; Low Density Lipoprotein, eGFR; Estimated Glomerular Filtration Rate.

^1^SBP <140 and DBP <90 mmHg.

^2^SBP ≥140 and/or DBP ≥ 90 mm Hg.

^3^SBP ≥140 and/or DBP ≥ 90 mm Hg with at least three antihypertensive medications regardless of class.

^4^ICD-code E10- E14.

^5^ICD-code I20-I25.

^6^ICD-code I48.

^7^ICD-code I50.

^8^ICD-code I61-I64.

^a^One way ANOVA.

^b^Pearson Chi-Square.

The proportion of individuals with documentation of alcohol consumption in the EMR (no documentation, PEth, alcohol history, or both) in relation to BP control, sex, age, and comorbidities is described in Table 2. More patients had a documented PEth value compared to a documented alcohol history. The type of alcohol documentation differed significantly across BP control groups, sex, age, and certain comorbidities (diabetes, atrial fibrillation, and congestive heart failure). Individuals with controlled hypertension were more likely to have both PEth and alcohol history documented, whereas those with uncontrolled hypertension or aTRH more often lacked documentation. Women were more likely than men to lack documentation of alcohol consumption in the EMR Younger individuals and those without diabetes were more likely to have PEth results recorded compared with older patients and individuals with diabetes.

**Table 2.** Documentation of Alcohol in Electronic Medical Records, in Relation to Blood Pressure Control, Sex, Age, and Comorbidities (2021-2023)

​	No documentation	PEth 16:0/18:1	Alcohol history^a^	PEth 16:0/18:1and alcohol history^a^	Total	P-value^b^
All % (n)	28.9 (527)	31.2 (570)	15.1 (276)	24.8 (453)	100.0 (1,826)	​
Controlled hypertension^1^ %(n)	24.1 (152)	31.2 (197)	15.3 (97)	29.4 (186)	100.0 (632)	0.00
Uncontrolled hypertension^2^% (n)	31.4 (285)	32.9 (299)	13.4 (122)	22.3 (203)	100.0 (909)	​
Treatment resistant hypertension^3^ % (n)	31.6 (90)	26.0 (74)	20.0 (57)	22.5 (64)	100.0 (285)	​
Women % (n)	34.4 (324)	31.6 (297)	13.5 (127)	20.5 (193)	100.0 (941)	0.00
Men % (n)	22.9 (203)	30.8 (273)	16.8 (149)	29.4 (260)	100.0 (885)	​
Age under 65 % (n)	22.4 (119)	33.7 (179)	16.8 (89)	27.1 (144)	100.0 (531)	0.00
Age 65 and over % (n)	31.5 (408)	30.2 (391)	14.4 (187)	23.9 (309)	100.0 (1,295)	​
Diabetes^4^ % (n)	29.7 (132)	8.6 (38)	34.2 (152)	27.5 (122)	100.0 (444)	0.00
Coronary artery disease,^5^ %(n)	31.3 (95)	25.7 (78)	17.8 (54)	25.3 (77)	100.0 (304)	0.11
Atrial fibrillation,^6^ % (n)	23.6 (65)	34.4 (95)	15.9 (44)	26.1 (72)	100.0 (276)	0.02
Congestive heart failure,^7^ % (n)	37.6 (71)	23.3 (44)	18.5 (35)	20.6 (39)	100.0 (189)	0.01
Cerebrovascular injury,^8^ % (n)	38.5 (57)	25.0 (37)	14.2 (21)	22.3 (33)	100.0 (148)	0.05

^1^SBP <140 and DBP <90 mmHg.

^2^SBP ≥140 and/or DBP ≥ 90 mm Hg.

^3^SBP ≥140 and/or DBP ≥ 90 mm Hg with at least three antihypertensive medications regardless of class.

^4^ICD-code E10- E14.

^5^ICD-code I20-I25.

^6^ICD-code I48.

^7^ICD-code I50.

^8^ICD-code I61-I64.

^a^Free text and/or AUDIT.

^b^Chi-square tests.

Among the three BP control groups, there was no statistically significant difference in the proportion of patients with hazardous alcohol, neither with PEth or alcohol history. Overall, the proportion of patients with indications of hazardous alcohol use was more than twice as high when measured by PEth (18.4%) compared to alcohol history (7.1%). Compared with women, men had a higher prevalence of hazardous alcohol use by PEth (25.2% vs 10.9%; p<0.001), whereas no significant sex difference was observed when based on alcohol history in the EMR (8.0% vs 6.0%; p=0.31).

## Discussion

This observational cross-sectional study examined alcohol documentation, using PEth and alcohol history, in a hypertensive population served by a primary care centre routinely applying PEth testing. Most individuals with hypertension had their alcohol use documented through PEth, alcohol history, or both within the past two years, with PEth recorded more frequently than alcohol history. Documentation patterns varied by BP control, age, sex, and comorbidities. No significant differences in the proportion of hazardous alcohol use were observed across the three BP control groups using either PEth or alcohol history.

Previous studies investigating alcohol habits among patients with hypertension have predominantly relied only on validated screening questionnaires such as the AUDIT questionnaire or unstructured medical history^20^ and not alcohol biomarkers. However, self-reporting of alcohol consumption carries a risk of underreporting,^21^ potentially hampering brief interventions for hazardous alcohol use.^22^ The findings that the proportion of hazardous alcohol use was more than twice as high with PEth^17^ compared to alcohol history may be due to the risk of patients underreporting alcohol consumption or as a result of different patient groups being tested with each method, possibly in different situations and for varying indications. The proportions of patients assessed with the different methods varied by sex, age, and comorbidities. This, may have contributed to the differences observed in the patient group classified as having hazardous alcohol use between PEth and alcohol history. PEth was used more frequently among patients under the age of 65 compared with those older than 65. The reason for this may be due to the clinical assumption that older individuals may consume less alcohol, for example due to illnesses or medication treatments. Among patients with diabetes, the proportion with documented PEth was the lowest. The health centre explained that they initially used PEth routinely in patients with diabetes, but that this practice was discontinued as high PEth values were seldom found in that group.

Even though PEth levels vary between individuals despite similar alcohol intake,^9^ GPs perceive PEth as an eye-opener that provides a more accurate picture of alcohol consumption compared to AUDIT.^12^ Findings from the present study indicate that PEth testing is used more often compared to alcohol history, a pattern that aligns with earlier research suggesting that the introduction of PEth may reduce reliance on alcohol history-taking.^11^ However, in the present study, both PEth and alcohol histories were documented in about a quarter of patients, in line with previous knowledge.^23,24^

Alcohol increases BP in a dose-dependent manner,^25^ and in a recent observational study,^10^ PEth measurements revealed substantially higher alcohol consumption in the group with aTRH compared with those with controlled and uncontrolled hypertension.^10^ However, in the present study there were no significant differences between prevalence of hazardous alcohol use between the three BP control groups regardless of method. Our findings though, must be interpreted with caution as individuals with uncontrolled BP and aTRH did not get their alcohol consumption documented to the same extent as those with controlled hypertension, potentially obscuring a true association. Several factors may further explain the lack of observed association. First, the study was not powered to detect differences in hazardous alcohol use across BP groups, which may have limited the ability to identify significant differences in the associations. Second, owing to the observational design, information on interventions for hazardous alcohol use was unavailable, and temporal relationships between alcohol history and PEth, and BP values, could not be established. Third, BP measurements were obtained from routine clinical practice without standardized measurement protocols, which may have introduced measurement variability. Fourth, data on medication adherence were not available, although this is a key determinant of BP control and may confound the relationship between alcohol use and hypertension outcomes. Fifth, because certain patient groups, such as individuals older than 80 years, were not routinely tested with PEth, and patients with diabetes were also frequently excluded from routine testing, questions arise regarding the representativeness and validity of the study population. Consequently, selection bias is probable. Furthermore, there may have been additional aspects of the primary care centre’s clinical routines, patient management practices, or other unmeasured factors that influenced testing patterns and, thereby, the study outcomes.

This study also showed that many individuals with hypertension failed to reach target BP despite treatment with three or more antihypertensive agents, aligning with previous findings.^26^

The present study did not collect data on how many individuals declined PEth testing and to what extent the GPs perceived PEth testing as problematic. The use of objective alcohol biomarkers require ethical considerations^27^ and GPs lack guidelines concerning PEth testing in relation to ethical dilemmas.^11^ However, discussions with the centre revealed that patients’ autonomy was respected when they refused testing. One might also speculate that, given that PEth has been used routinely for nearly a decade at the centre, ethical concerns should not argue against its continued routine use as the use of PEth is increasing in health care,^9,28^ including primary care.^12,29^ Additionally, one should bear in mind that PEth is not only employed to identify hazardous alcohol use, but also to clarify low levels of alcohol consumption.^30^

A strength of this study is the inclusion of nearly all patients with diagnosed hypertension within one primary care population, enhancing the internal validity of findings on PEth and alcohol history documentation. However, several limitations apply. The single-site, non-randomized, and relatively small sample limits generalizability. The local PEth testing routine, systematic for most patients but excluding those >80 years and many with diabetes unless previously elevated PEth levels, likely contributed to the lower documentation rates in these groups. PEth testing was also introduced pragmatically in response to low AUDIT completion and implemented without a formal protocol, which may have introduced variation. Importantly, the PEth routine was not designed or managed by the research team; thus, the data reflect real-world practice rather than a controlled intervention.

The utilisation of PEth is increasing within healthcare,^9^ partly owing to its 100% specificity for ethanol exposure.^27^ Although interpretation guidelines are available,^8,9^ it is important to acknowledge that inter-individual variability influences the magnitude of PEth concentrations.^9^ Consequently, repeated PEth measurements in the same patient may offer additional clinically relevant information. As PEth analysis is a comparatively costly laboratory test, further research evaluating its cost-effectiveness is warranted.

The threshold used to define controlled hypertension is not consistent with current US^31^ or European^2^ guideline recommendations, but reflects a pragmatic classification derived from the clinical quality system from which the data were obtained. The validity of the BP measurements may be questioned, as they were documented without information on measurement methods or technique, limiting their accuracy. BP values recorded in EMRs only partly correspond to those obtained with validated methods.^26^ However, in primary care, clinicians often rely on pragmatic thresholds rather than strictly applying US or European guidelines,^32^ making the <140/90 mmHg cut-off relevant to the study context. Because BP, PEth, and alcohol history were documented at unknown times across a two-year period, temporal relationships cannot be established. Finally, the study lacked sociodemographic data such as education and income, which may influence alcohol use and hypertension management.

## Conclusions

This study shows that PEth testing can be integrated into routine primary care as a complement to free text alcohol history and AUDIT in patients with hypertension.

Elevated PEth levels were observed across both sexes and all BP control categories, supporting the potential clinical value of PEth testing. While the impact on detecting hazardous use or improving BP control cannot be determined, combining objective and subjective methods may strengthen clinicians’ evaluation of alcohol related risk.

Routine PEth testing also raises ethical considerations regarding consent, underscoring the need for further research on implementation and patient experiences.

## Data Availability

The data from this cohort are available upon reasonable request. Data access is governed by data sharing agreements that comply with relevant privacy and ethical guidelines.*

## Appendix

### List of Abbreviations

AUDIT: Alcohol Use Disorders Identification Test
BMI: Body Mass Index
BP: Blood Pressure
CNI: Care Need Index
eGFR: Estimated Glomerular Filtration Rate
EMR: Electronic Medical Records
GP: General Practitioner
ICD-10: International Classification of Diseases, Tenth Revision
LC–MS/MS: Liquid Chromatography–Tandem Mass Spectrometry
LDL: Low-Density Lipoprotein
M: Mean
PEth: Phosphatidylethanol
SD: Standard Deviation
aTRH: Apparent Treatment Resistant Hypertension.
